# Systematic Literature Review (SLR) and Network Meta-Analysis (NMA) of First-Line Therapies (1L) for Locally Advanced/Metastatic Urothelial Carcinoma (la/mUC)

**DOI:** 10.3390/curroncol30040277

**Published:** 2023-03-26

**Authors:** Lisa Bloudek, Phoebe Wright, Caroline McKay, Christina Louise Derleth, Jennifer Susan Lill, Enrique Lenero, Zsolt Hepp, Scott David Ramsey, Sean D. Sullivan, Beth Devine

**Affiliations:** 1 Curta Inc., Seattle, WA 98116, USA; 2 Seagen Inc., Bothell, WA 98021, USA; 3 Astellas Pharma Global Development, Inc., Northbrook, IL 60062, USA; 4 CHOICE Institute, School of Pharmacy, University of Washington, Seattle, WA 98195, USA; 5 Hutchinson Institute for Cancer Outcomes Research, Fred Hutchinson Cancer Center, Seattle, WA 98109, USA

**Keywords:** bladder cancer, systematic literature review, network meta-analysis, standard of care, oncology, overall survival

## Abstract

To compare efficacy outcomes for all approved and investigational first-line (1L) treatment regimens for locally advanced or metastatic urothelial carcinoma (la/mUC) with standard of care (SOC), a network meta-analysis (NMA) was conducted. A systematic literature review (SLR) identified phase 2 and 3 randomized trials investigating 1L treatment regimens in la/mUC published January 2001–September 2021. Three networks were formed based on cisplatin (cis) eligibility: cis-eligible/mixed (cis-eligible patients and mixed populations of cis-eligible/ineligible patients), cis-ineligible (strict; exclusively cis-ineligible patients), and cis-ineligible (wide; including studies with investigator’s choice of carbo). Analyses examined comparative efficacy by hazard ratio (HR) for overall survival (OS), and progression-free survival (PFS), and odds ratio (OR) for overall response rate (ORR), with 1L regimens vs. SOC. SOC was gemcitabine + cis (GemCis) or carboplatin (GemCarbo), cis-eligible/mixed network, and GemCarbo cis-ineligible networks. Of 1906 SLR identified citations, 55 trials were selected for data extraction. The NMA comprised 11, 6, and 8 studies in the cis-eligible/mixed, cis-ineligible (strict), cis-ineligible (wide) networks, respectively. In a meta-analysis of SOC control arms, median (95% CI) overall survival (OS) in months varied by network: 13.19 (12.43, 13.95) cis-eligible/mixed, 11.96 (10.43, 13.48) cis-ineligible (wide), and 9.74 (6.71, 12.76) cis-ineligible (strict). Most differences in OS, PFS, and ORR with treatment regimens across treatment networks were not statistically significant compared with SOC. Outcomes with current 1L regimens remain poor, and few significant improvements over SOC have been made, despite inclusion of recent clinical trial data, highlighting an unmet need in the la/mUC patient population.

## 1. Introduction

Locally advanced or metastatic urothelial carcinoma (la/mUC) is an aggressive disease with a 5-year survival rate of 5–7% [1,2]. The National Comprehensive Cancer Network (NCCN) and European Society for Medical Oncology (ESMO) guideline recommendation for first-line (1L) treatment of la/mUC is gemcitabine (Gem) in combination with a platinum-based agent (GemPlat), either cisplatin (cis (GemCis)) or Gem in combination with carboplatin (carbo (GemCarbo)) for patients not eligible for cis due to renal impairment, congestive heart failure, poor performance status, or other reasons [3]. Avelumab maintenance therapy has recently been recommended for the subset of patients with la/mUC whose disease has not progressed following 1L platinum-based therapy [3,4].

Long-term survival with standard of care (SOC) remains limited, even more so in the ~50% of patients with la/mUC who are ineligible for cis-based treatment [5]. The complexity of the treatment landscape has increased since the introduction of the programmed cell death protein 1/ligand 1 (PD-1/PD-L1) checkpoint inhibitors (e.g., atezolizumab and pembrolizumab) that are recommended as alternative treatment options in patients with PD-L1-expressing tumors and who are not eligible for cis or in patients who are unable to receive cis or carbo treatment, regardless of the PD-L1 status of their tumors [3]. Due to continued suboptimal outcomes with SOC, 1L treatment of la/mUC is an area of ongoing innovation and evaluation of novel treatment regimens, with new clinical trials that aimed to improve on SOC having been completed in recent years.

The objective of this study was to conduct a systematic literature review (SLR) of all approved and investigational 1L regimens from phase 2/3 randomized controlled trials (RCTs) in patients with la/mUC to compare the efficacy outcomes of these regimens with SOC through a network meta-analysis (NMA). The NMA enabled an indirect treatment comparison of survival outcomes with different 1L treatments for la/mUC from multiple studies.

## 2. Materials and Methods

### 2.1. Search Strategy

An SLR to evaluate data on the efficacy and safety of 1L treatment regimens in patients with la/mUC from RCTs was conducted. SLR methods used were in accordance with the Preferred Reporting Items for Systematic Reviews and Meta-Analyses (PRISMA) and National Institute for Clinical Excellence (NICE) Decision Support Unit guidelines.

EMBASE and MEDLINE (via PubMed) were searched to identify references reporting results from phase 2 and 3 RCTs that investigated systemic therapy in la/mUC, published after January 2000 and before September 2021.

To capture all relevant data, electronic searches were supplemented with hand-searching of the proceedings of relevant scientific conferences and health technology assessment (HTA) submissions from 2015 to 2021. HTA organizations included NICE, Haute Autorité de Santé (HAS), Institute for Quality and Efficiency in Health Care (IQWiG), and Scottish Medicines Consortium (SMC). Scientific conferences included the American Association for Cancer Research (AARC), the American Society of Clinical Oncology (ASCO), ASCO Genitourinary Cancers Symposium (ASCO-GU), ESMO, and Society of Urologic Oncology (SUO). References were screened for inclusion or exclusion by 2 independent investigators, with a third investigator consulted when necessary to reach consensus.

### 2.2. Inclusion and Exclusion Criteria

RCTs were included if they investigated 1L treatment in la/mUC and reported data on overall survival (OS), progression-free survival (PFS), or overall response rate (ORR). Single-arm studies of PD-1/PD-L1 checkpoint inhibitors, single-arm studies in a cis-ineligible population, and previously published SLRs and NMAs were included in the SLR as background information on SOC. The minimum sample size for inclusion was n ≥ 15 in each study arm and non-English language publications were excluded.

The quality of included studies was assessed by a single investigator using the Cochrane Collaboration risk of bias in randomized controlled trials version 2 (RoB2) tool (Supplementary Table S1).

### 2.3. Meta-Analysis Methodology

A meta-analysis was conducted to generate point estimates and 95% confidence intervals for median OS, median PFS, and ORR for SOC and across all 1L treatment regimens. A Bayesian network meta-analysis with uninformed priors was conducted to assess the comparative efficacy of OS, PFS, and ORR with 1L la/mUC regimens vs. SOC. NMA methodology followed NICE decision support unit and ISPOR guidelines for conducting NMAs using data from RCTs [6,7].

Three networks were formed: 1. a cis-eligible/mixed network comprising studies that recruited a strictly cis-eligible population and the overall study results from studies that recruited both patients eligible and ineligible for cis, with investigator’s choice of platinum chemotherapy, in order to include contemporary trials that have included a mixed patient population [8,9,10]. 2. A cis-ineligible (wide) criteria network that included studies of patients who were cis-ineligible and was expanded to add studies where a subgroup was presented based on the investigator’s choice of carbo, regardless of cis eligibility or ineligibility according to the Galsky criteria [5]. 3. A cis-ineligible (strict) network that included studies that exclusively recruited patients who were cis-ineligible or presented data for the cis-ineligible subgroup, with cis ineligibility guided by the Galsky criteria. SOC was defined as GemPlat (GemCis or GemCarbo) in the cis-eligible/mixed network and GemCarbo in the cis-ineligible networks. Maintenance studies were not included in the NMA, as their design assessed outcomes from time of randomization to maintenance rather than initiation of 1L treatment.

Results are presented in network diagrams in which each color used represents a particular treatment. When multiple treatments are used in combination, the color of the first treatment is used. A fixed effect model was used in each analysis, as heterogeneity between studies was acceptable according to Cochrane’s Q and Higgin’s I2 criteria [11].

## 3. Results

### 3.1. Study Selection and Characteristics

Electronic database searches identified 2340 publications, which were supplemented with 275 hand-searched references. In total, 1906 citations underwent title and abstract screening, of which 315 were screened at full-text. Overall, 163 references reporting data from 55 trials were selected for data extraction. The PRISMA diagram of the results of all searches is shown in Supplementary Figure S1.

### 3.2. Network Meta-Analysis

Of the 55 trials that were selected for data extraction in the SLR, 37 were excluded from the NMA; reasons for exclusion are summarized in Supplementary Figure S2. The NMA comprised 11 RCTs in the cis-eligible/mixed network, 8 RCTs in the cis-ineligible (wide) network, and 6 RCTs in the cis-ineligible (strict) network. The NMA included patients from the key recent phase 3 trials, including KEYNOTE-361 [8,12], IMvigor130 [9,13], and DANUBE [10] studies. An overview of the studies included in each treatment network is provided in Supplementary Table S2.

### 3.3. Overall Survival by Treatment Network

#### 3.3.1. Cis-Eligible/Mixed Network

**Figure 1:**
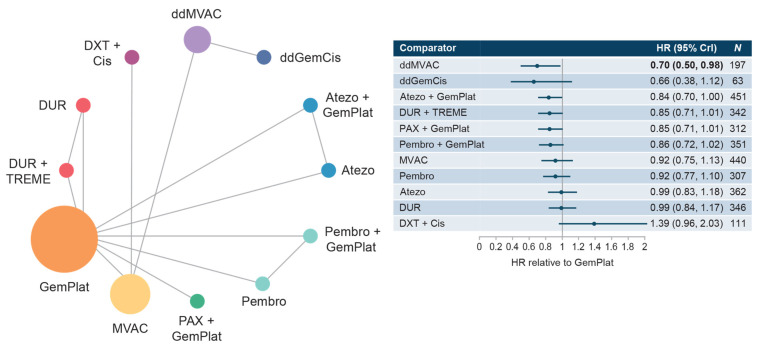
Network diagram and HR for OS by treatment network for the cis-eligible/mixed network. Abbreviations: Atezo, atezolizumab; cis, cisplatin; CrI, credible interval; ddGemCis, dose-dense gemcitabine + cisplatin; ddMVAC, dose-dense methotrexate + vinblastine + doxorubicin + cisplatin; DUR, durvalumab; DXT, docetaxel; GemPlat, gemcitabine + platinum (cisplatin or carboplatin); HR, hazard ratio; MVAC, methotrexate + vinblastine + doxorubicin + cisplatin; OS, overall survival; PAX, paclitaxel; Pembro, pembrolizumab; TREME, tremelimumab.

The cis-eligible/mixed network included six studies that reported OS data for SOC plus nine comparator treatment regimens. Median OS with SOC GemCis or GemCarbo (GemPlat) was 13.19 months (95% confidence interval [CI]: 12.43, 13.95) (Supplementary Figure S3). Hazard ratios (HRs) for treatment regimens compared with SOC ranged from 0.66 to 1.39 with no statistically significant differences from SOC for most regimens, including regimens that utilized PD-1/PD-L1 checkpoint inhibitors: atezolizumab + GemPlat (HR: 0.84; 95% credible intervaI [Crl]: 0.70, 1.00), durvalumab + tremelimumab (HR: 0.85; 95% CrI: 0.71, 1.01), and pembrolizumab monotherapy (HR: 0.92; 95% CrI: 0.77, 1.10). Significantly longer OS compared with SOC was shown only for dose-dense methotrexate + vinblastine + doxorubicin + cis (ddMVAC) (HR: 0.70; 95% CrI: 0.50, 0.98) (Figure 1). Few comparisons across regimens were statistically significantly different from each other (Supplementary Table S3).

#### 3.3.2. Cis-Ineligible (Wide) Network

**Figure 2:**
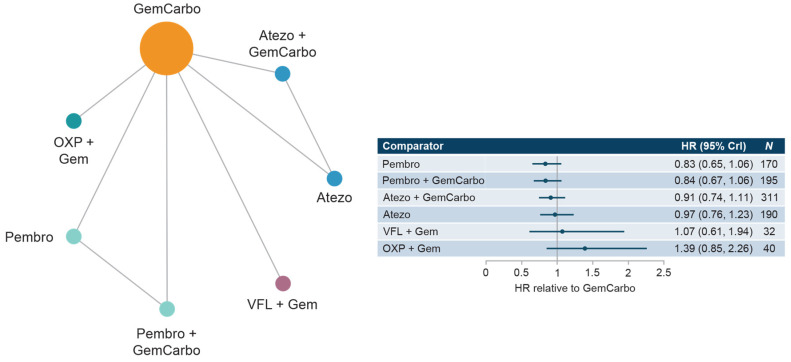
Network diagram and HR for OS by treatment network for the cis-ineligible (wide) network. Abbreviations: Atezo, atezolizumab; cis, cisplatin; CrI, credible interval; Gem, gemcitabine; GemCarbo, gemcitabine + carboplatin; HR, hazard ratio; OS, overall survival; OXP, oxaliplatin; Pembro, pembrolizumab; VFL, vinflunine.

The cis-ineligible (wide) network included six studies that reported OS data for SOC plus six comparator treatments. Median OS for SOC (GemCarbo) was 11.96 months (95% CI: 10.43, 13.48), Supplementary Figure S4. HRs compared with SOC ranged from 0.83 to 1.39 with no statistically significant differences in OS compared with SOC (Figure 2). Comparisons across regimens were not statistically significantly different from each other (Supplementary Table S4).

#### 3.3.3. Cis-Ineligible (Strict) Network

**Figure 3:**
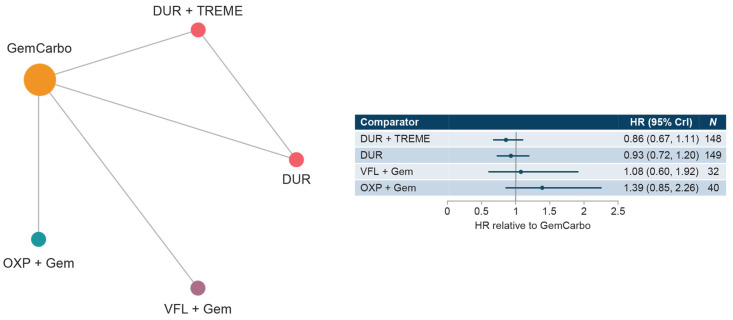
Network diagram and HR for OS by treatment network for the cis-ineligible (strict) network. Abbreviations: Cis, cisplatin; CrI, credible interval; DUR, durvalumab; Gem, gemcitabine; GemCarbo, gemcitabine + carboplatin; HR, hazard ratio; OXP, oxaliplatin; OS, overall survival; TREME, tremelimumab; VFL, vinflunine.

The cis-ineligible (strict) network included four studies that reported OS data for SOC plus four comparator treatment regimens. Median OS for SOC (GemCarbo) was 9.74 months (95% CI: 6.71, 12.76), Supplementary Figure S5. HRs compared with SOC ranged from 0.86 to 1.39 with no statistically significant differences in OS compared with SOC (Figure 3). Comparisons across regimens were not statistically significantly different from each other (Supplementary Table S5).

### 3.4. Progression-Free Survival by Treatment Network

#### 3.4.1. Cis-Eligible/Mixed Network

**Figure 4:**
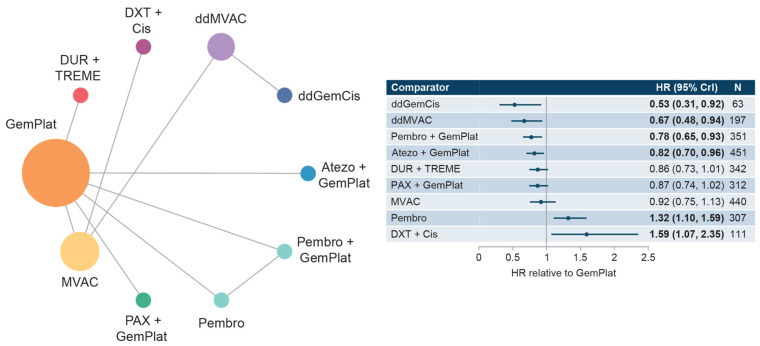
Network diagram and HR for PFS by treatment network for the cis-eligible/mixed network. Abbreviations: Cis, cisplatin; CrI, credible interval; ddGemCis, dose-dense gemcitabine + cisplatin; ddMVAC, dose-dense methotrexate + vinblastine + doxorubicin + cisplatin; DUR, durvalumab; DXT, docetaxel; GemPlat, gemcitabine + platinum (cisplatin or carboplatin); HR, hazard ratio; MVAC, methotrexate + vinblastine + doxorubicin + cisplatin; PAX, paclitaxel; Pembro, pembrolizumab; PFS, progression-free survival; TREME, tremelimumab.

This network included five studies that reported PFS data for SOC plus six comparator treatments. Median PFS for SOC (GemPlat) was 6.85 months (95% CI: 6.26, 7.44), Supplementary Figure S6. HRs compared with SOC ranged from 0.53 to 1.59 with significantly longer PFS reported for dose-dense GemCis (ddGemCis), ddMVAC, pembrolizumab + GemPlat, and atezolizumab + GemPlat. Significantly shorter PFSs were reported for pembrolizumab and docetaxel + cis (Figure 4). Few comparisons across regimens were statistically significantly different from each other (Supplementary Table S6).

#### 3.4.2. Cis-Ineligible (Wide) + Cis-Ineligible (Strict) Networks

**Figure 5:**
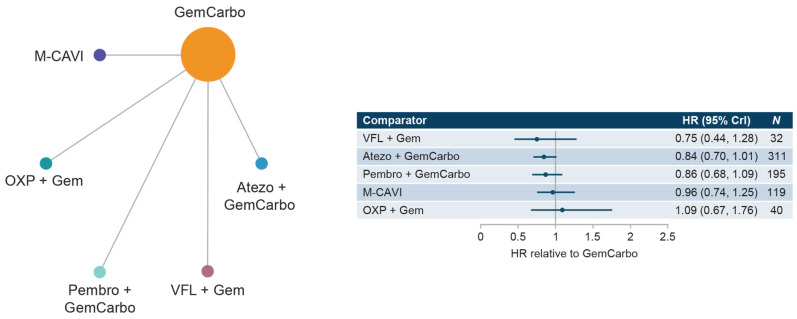
Network diagram and HR for PFS by treatment network for the cis-ineligible (wide) network. Abbreviations: Atezo, atezolizumab; cis, cisplatin; CrI, credible interval; Gem, gemcitabine; GemCarbo, gemcitabine + carboplatin; HR, hazard ratio; M-CAVI, methotrexate + carboplatin + vinblastine; OXP, oxaliplatin; Pembro, pembrolizumab; PFS, progression-free survival; VFL, vinflunine.

**Figure 6:**
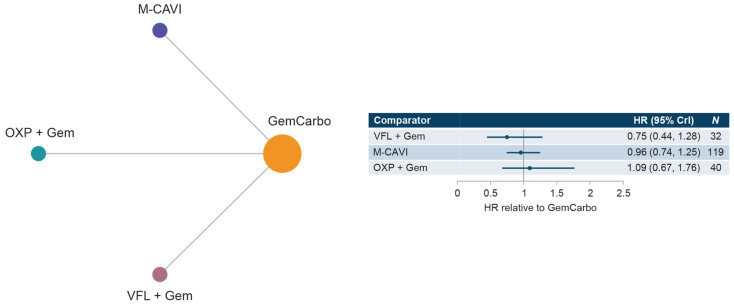
Network diagram and HR for PFS by treatment network for the cis-ineligible (strict) network. Abbreviations: CrI, credible interval; Gem, gemcitabine; GemCarbo, gemcitabine + carboplatin; HR, hazard ratio; M-CAVI, methotrexate + carboplatin + vinblastine; OXP, oxaliplatin; PFS, progression-free survival; VFL, vinflunine.

Three studies were included in the overall cis-ineligible network that reported PFS data for SOC plus three comparator treatments. Median PFS for SOC (GemCarbo) was 5.61 months (95% CI: 4.95, 6.26) (Supplementary Figure S7) for both the cis-ineligible (wide) and cis-ineligible (strict) networks. HRs compared with SOC ranged from 0.75 to 1.09 in both the cis-ineligible (wide) and cis-ineligible (strict) networks, and no treatment regimen was shown to result in a statistically significant difference in PFS compared with SOC in either network (Figure 5 and Figure 6). Comparisons across regimens were not statistically significantly different from each other (Supplementary Tables S7 and S8).

### 3.5. Overall Response Rate by Treatment Network

The cis-eligible/mixed, cis-ineligible (wide) and cis-ineligible (strict) networks included 11, 8, and 6 studies, respectively, which reported ORR data for SOC plus 13, 8, and 7 treatments. For SOC (GemPlat, GemCis, and GemCarbo respectively), the ORR was 46% (95% CI: 44, 49%) for cis-eligible/mixed, 42% (95% CI: 39, 46%) for cis-ineligible (wide), and 42% (95% CI: 37, 48%) for cis-ineligible (strict). Significantly higher ORR compared with SOC was seen in the cis-eligible/mixed network for paclitaxel + GemPlat (odds ratio (OR): 1.51; 95% Crl: 1.13, 2.03) and pembrolizumab + GemPlat (OR: 1.49; 95% Crl: 1.11, 2.01) (Supplementary Table S2).

## 4. Discussion

Long-term survival in patients with la/mUC is limited [3], and this study demonstrates that, despite recent trials investigating alternative 1L treatment regimens, no clear improvements in OS when compared with SOC have been observed. While survival may be relatively longer in patients treated with GemCis, compared with GemCarbo, outcomes in all patients are poor and many patients are ineligible for cis-based treatment [5,14]. The inclusion of three different networks enabled analysis of a broader population, including contemporary clinical trials—KEYNOTE-361 [8], IMvigor 130 [9], and DANUBE [10]—that reported a mixed population of cis-eligible and cis-ineligible patients, while also reporting subgroup results from exclusively cis-eligible and cis-ineligible patients or from the subgroup with the investigator’s choice of carbo.

It was not possible to include studies of maintenance therapies in these analyses due to differences in study design compared with trials of 1L treatments. The JAVELIN trial of maintenance avelumab assessed overall survival from initiation of maintenance therapy among the subset of patients who had not progressed following 1L GemPlat treatment, rather than from the start of 1L therapy [15]. Given the difference in OS endpoint assessment between 1L treatment trials and maintenance trials and the criteria for this study, assessments of maintenance were outside of the scope of these analyses.

OS in the cis-eligible/mixed network was similar to SOC (13.19 months) across all interventions, except for ddMVAC, and remained poor among established and recently evaluated therapies in 1L la/mUC, despite inclusion of recent trial data for the emergent PD-1/PD-L1 checkpoint inhibitors. This result is consistent with the notably long OS (median 15.2 months) reported in a study of MVAC included in this analysis [16]. However, that study enrolled patients with factors indicative of a better prognosis, such as adequate renal function, ECOG 0 or 1, absence of visceral metastases, and a younger population of patients compared with other trials. Additionally, median OS in the cis-ineligible (strict) network with standard of care GemCarbo (9.74 months) was similar to the 8.39 months reported in a prior NMA of 1L treatments for cis-ineligible patients with la/mUC [17]. OS was similar to SOC in most comparisons in this analysis, which was expected given that few recent 1L studies have shown improved survival over SOC [8,9,12,18]. This also aligns with a prior NMA that included three PD-1/PD-L1 checkpoint inhibitor therapy studies in patients with mUC, which found that OS with PD-1/PD-L1 checkpoint inhibitor therapy monotherapy or combined with platinum-based chemotherapy was not superior to SOC [19]. In contrast, in a separate NMA of the same PD-1/PD-L1 checkpoint inhibitor therapy studies by Mori et al. 2021, OS was significantly greater in patients treated with PD-1/PD-L1 checkpoint inhibitor therapy combined with platinum-based chemotherapy compared with platinum-based chemotherapy alone; however, the CrI approached 1 for this outcome (HR: 0.85, 95% CrI: 0.76, 0.94) [20]. Mori et al. 2021 also pooled outcomes with PD-1/PD-L1 checkpoint inhibitor therapies, rather than assessing each separately, as in this analysis [20]. In addition, recent data from the IMvigor 130 trial reported that mOS outcomes are similar following treatment with either GemCis or GemCarbo (13.4 [95% CI 11.7–18.4] versus 13.4 [95% CI 10.8–15.6]), suggesting that choice of 1L SOC treatment may have limited impact on patient outcomes. [21]

PFS with each treatment regimen was also broadly similar to SOC, with no significant differences in the cis-ineligible networks; however, in the cis-eligible/mixed network, we observed more variation, with significant differences in PFS (both improved and shorter) among the different treatment regimens. These results should also be interpreted with caution, as some treatment regimens that did not improve PFS, such as PD-1/PD-L1 checkpoint inhibitor monotherapy, did not report HRs or confidence intervals, which may have resulted in reporting bias within the cis-eligible/mixed network. As discordance between PFS and OS results is not uncommon [22], OS is typically accepted as the most meaningful treatment outcome [23].

Analysis of ORR showed that the majority of patients, regardless of treatment network (cis-eligible/mixed, cis-ineligible (wide), or cis-ineligible (strict)) did not have a meaningful change in response compared with SOC or any treatment regimen. This is notable because previous clinical trials have reported ORRs for treatments including ddMVAC and oxaliplatin + gem that exceed those seen with SOC [24,25]. Additionally, both this study and a previous NMA in this indication found that ORR was greatest in cis-eligible la/mUC patients given paclitaxel + GemPlat [26]. In contrast, ORRs with PD-1/PD-L1 inhibitor monotherapy (24–28%) [10,12] tended to be lower than with SOC in the cis-ineligible network in this study (42%).

With the exception of PD-1/PD-L1 inhibitors, the therapeutic landscape for 1L treatment in la/mUC has changed little and 1L platinum-based therapy remains the SOC. However, clinical trials investigating the use of novel therapeutic agents with innovative mechanisms of action are ongoing. For example, the EV-302 trial is investigating the efficacy of the Nectin-4 targeted antibody–drug conjugate enfortumab vedotin. In the EV-103 Phase 1b/2 trial in cisplatin-ineligible patients with previously untreated la/mUC (randomized Cohort K), enfortumab vedotin in combination with pembrolizumab demonstrated a clinically meaningful ORR of 64.5%, as did enfortumab vedotin monotherapy (ORR, 45.2%) [27].

### Limitations

Networks were primarily constructed of single connections and evidence was rarely available for a regimen from multiple studies other than for SOC. Heterogeneity across studies is a significant limitation, with outcome ranges for SOC treatments varying substantially between treatment networks. This heterogeneity may indicate differences within patient populations in the included RCTs, but attempts to adjust for these differences across studies were unsuccessful because of the limited number of studies available for each treatment regimen. Differences in design between studies of maintenance therapies and those for 1L treatments, particularly around the point from which OS is assessed, meant that it was not possible to include maintenance studies in these analyses. Lastly, although studies included some discussion of adverse events, insufficient data were available to include safety as an outcome in the NMA.

## 5. Conclusions

While there are a number of limitations associated with this NMA, it is the most up-to-date NMA conducted for 1L therapies in patients with la/mUC. The data suggest that there have been only modest advances in treatment benefit with alternative 1L regimens. Outcomes with recently investigated regimens, including the PD-1/PD-L1 checkpoint inhibitor monotherapies atezolizumab, durvalumab, and pembrolizumab, were broadly similar to SOC, irrespective of cis eligibility. These results highlight the continued unmet need for novel, effective, and safe 1L treatments that improve survival in patients with la/mUC.

## Data Availability

Data are contained within the article and Supplementary Materials.

## Supplementary Materials

The following supporting information can be downloaded at: https://www.mdpi.com/article/10.3390/curroncol30040277/s1, Figure S1: PRISMA Diagram showing all searches performed in the SLR [28,29,30,31,32,33,34,35,36]; Figure S2: Study identification and attrition for the NMA; Figure S3: Median OS for SOC (GemPlat) in the cis-eligible/mixed network [37,38]; Figure S4: Median OS for SOC (GemCarbo) in the cis-ineligible (wide) network [39]; Figure S5: Median OS for SOC (GemCarbo) in the cis-ineligible (strict) network; Figure S6: Median PFS for SOC (GemPlat) in the cis-eligible/mixed network; Figure S7: Median PFS for SOC (GemCarbo) in the overall cis-ineligible networks. Table S1: Cochrane risk-of-bias tool for randomized trials (RoB2); Table S2: Overview of studies in each network [25]; Table S3: League table of relative treatment effects on OS (HR, Crl). Cis-eligible/mixed network; Table S4: League table of relative treatment effects on OS (HR, Crl). Cis-ineligible (wide) network; Table S5: League table of relative treatment effects on OS (HR, Crl). Cis-ineligible (strict) network; Table S6: League table of relative treatment effects on PFS (HR, Crl), Cis-eligible/mixed; Table S7: League table of relative treatment effects on PFS (HR, Crl). Cis-ineligible (wide) network; Table S8: League table of relative treatment effects on PFS (HR, Crl). Cis-ineligible (strict) network. Additional references [28,29,30,31,32,33,34,35,36,37,38,39].
