# Supplementary material for: Systematic Literature Review (SLR) and Network Meta-Analysis (NMA) of First-Line Therapies (1L) for Locally Advanced/Metastatic Urothelial Carcinoma (la/mUC)
Source: Curr Oncol. 2023 Mar 26;30(4):3637–47. doi: 10.3390/curroncol30040277 (PMC10136539; doi:10.3390/curroncol30040277)
Supplement: Supplementary file 1 [file curroncol-30-00277-s001.zip › curroncol-2234267-supplementary.pdf]

Supplementary Materials

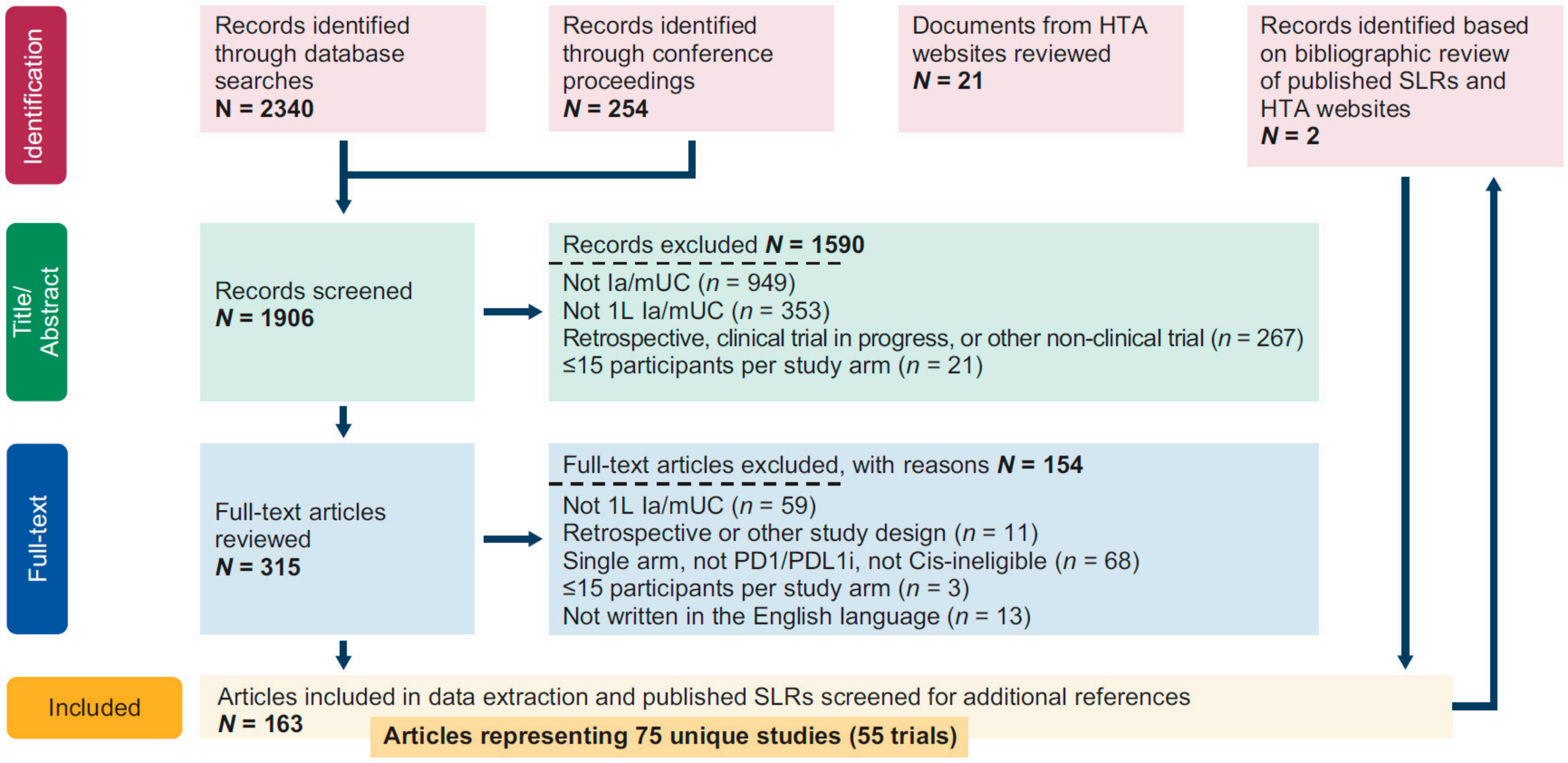

Supplementary Figure S1. PRISMA Diagram showing all searches performed in the SLR.
1L, first-line; la/mUC, locally advanced or metastatic urothelial carcinoma; Cis, cisplatin; HTA, health technology assessment; PD1, programmed death-1; PDL1i, programmed death-ligand 1 inhibitor; PRISMA, preferred reporting items for systematic reviews and meta-analyses; SLR, systematic literature review.

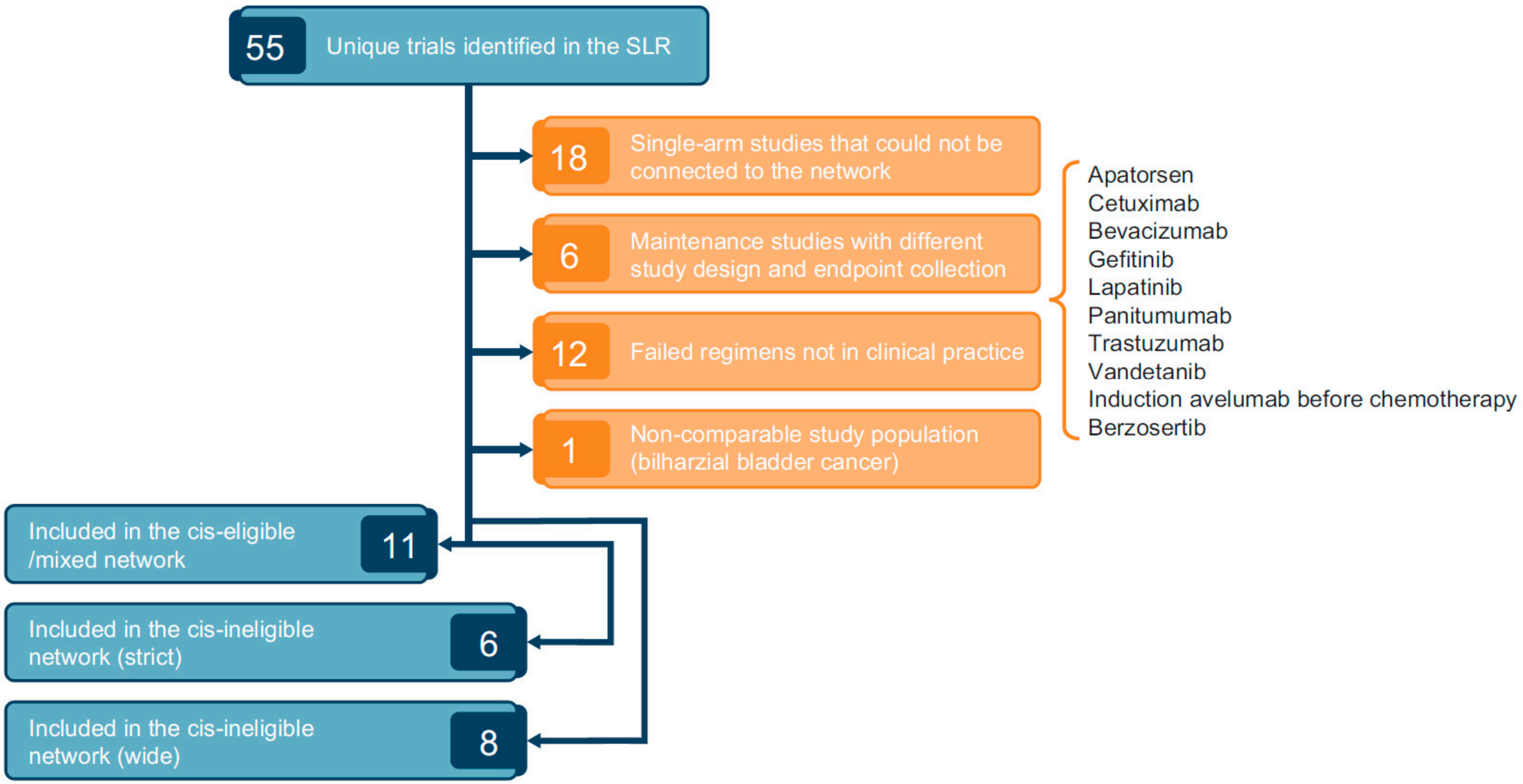

Supplementary Figure S2. Study identification and attrition for the NMA.
Cis, cisplatin; NMA, network meta-analysis; SLR, systematic literature review.

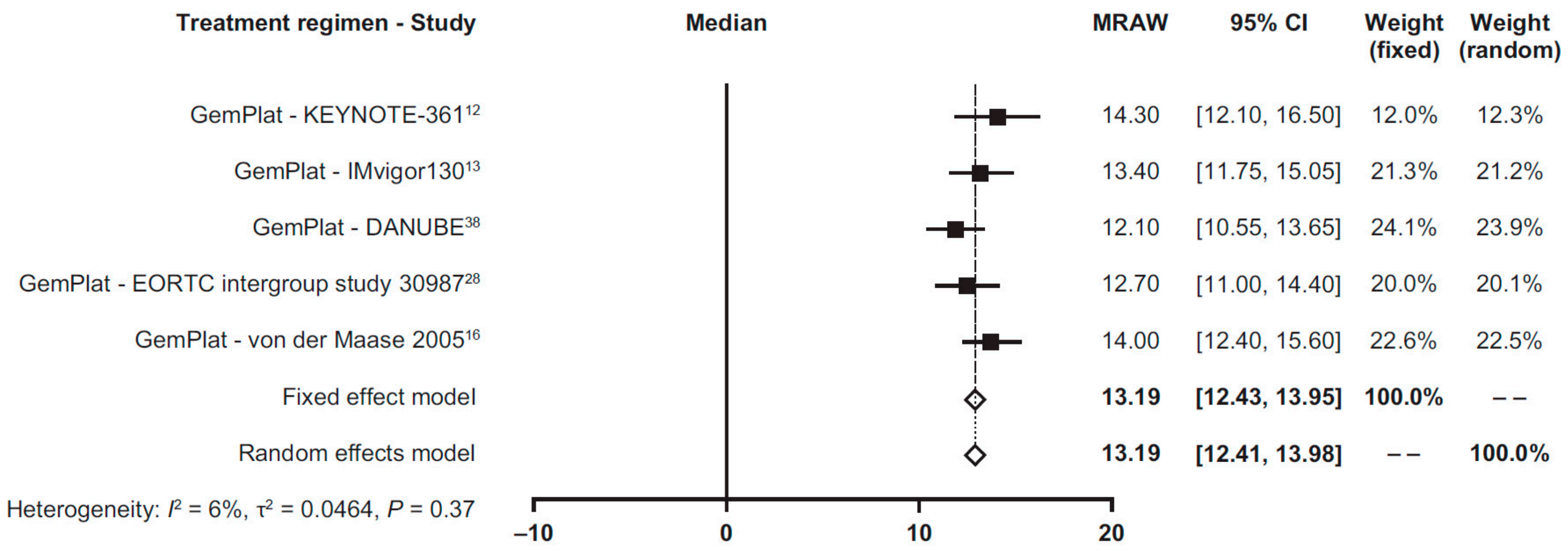

Supplementary Figure S3. Median OS for SOC (GemPlat) in the cis-eligible/mixed network.

References = [12,13,16,28,38]

CI , confidence interval; Cis, cisplatin; GemPlat, gemcitabine + platinum (cisplatin or carboplatin); MRAW, raw or untransformed mean; OS, overall survival; SOC, standard of care.

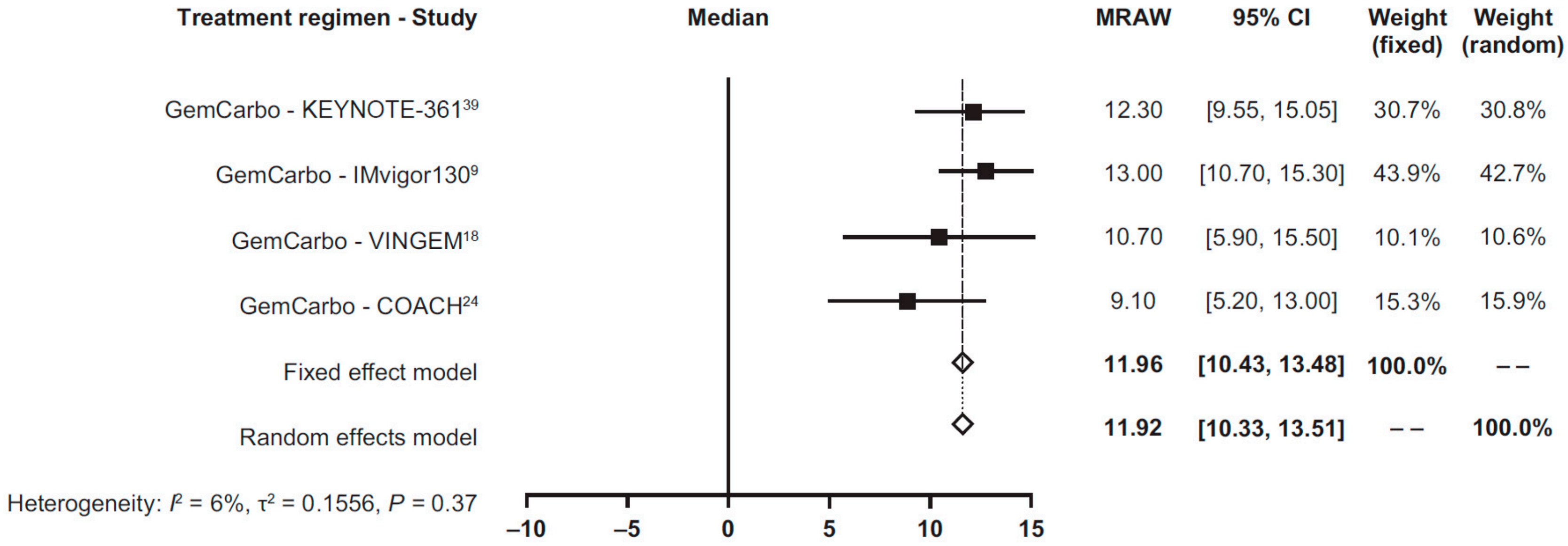

Supplementary Figure S4. Median OS for SOC (GemCarbo) in the cis-ineligible (wide) network.

References = [9,18,24,39]

CI, confidence interval; Cis, cisplatin; GemCarbo, gemcitabine + carboplatin; MRAW, raw or untransformed mean; OS, overall survival; SOC, standard of care.

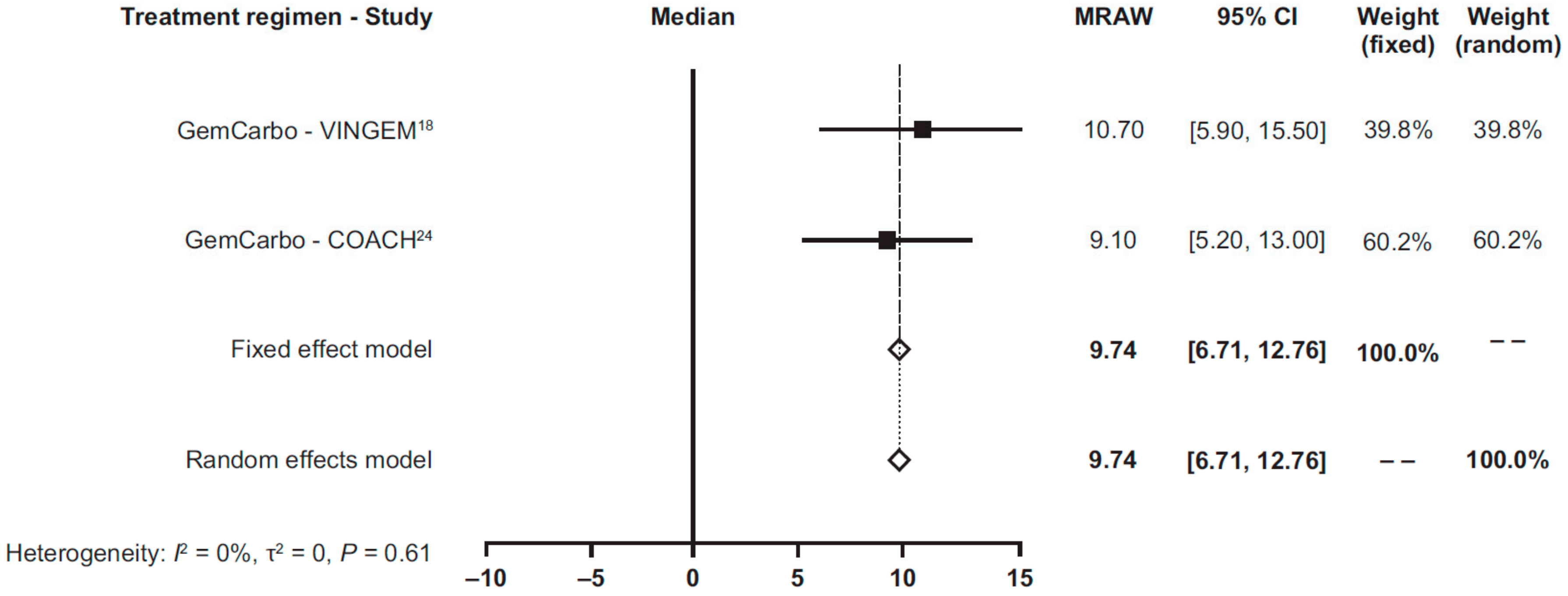

Supplementary Figure S5. Median OS for SOC (GemCarbo) in the cis-ineligible (strict) network.
References = [18,24]
CI, confidence interval; Cis, cisplatin; GemCarbo, gemcitabine + carboplatin; MRAW, raw or untransformed mean; OS, overall survival; SOC, standard of care.

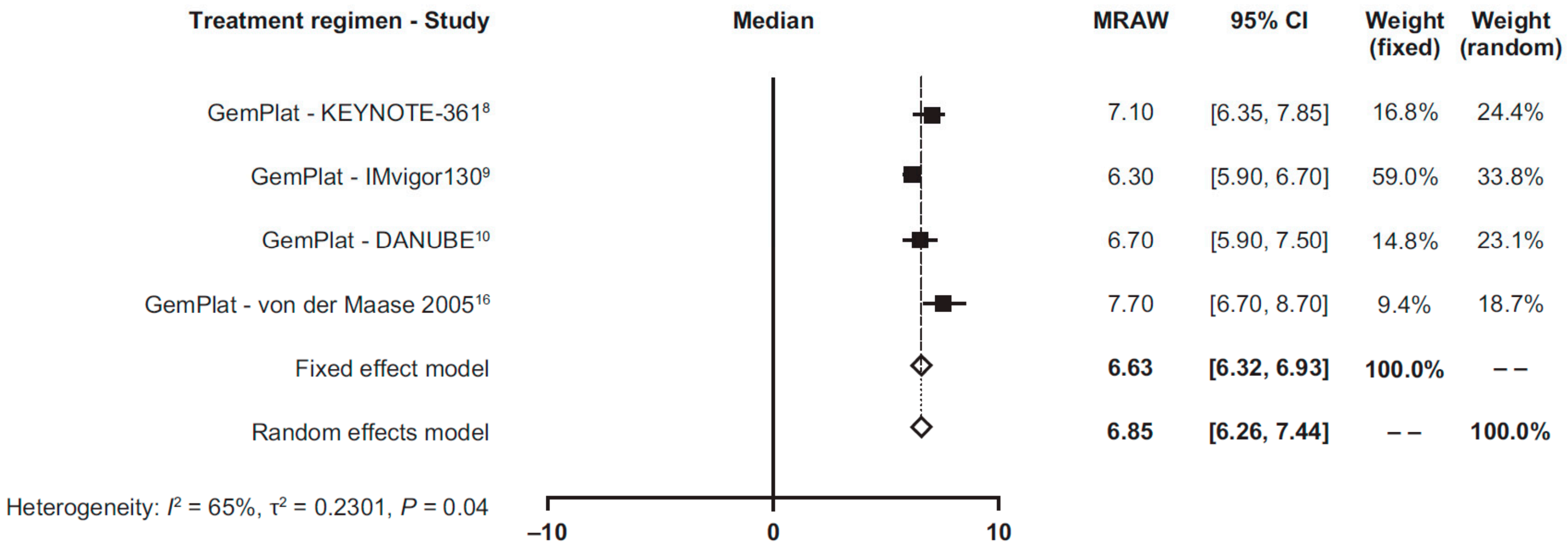

Supplementary Figure S6. Median PFS for SOC (GemPlat) in the cis-eligible/mixed network

References = [8,9,10,16]

CI, confidence interval; Cis, cisplatin; GemPlat, gemcitabine + platinum (cisplatin or carboplatin); MRAW, raw or untransformed mean; OS, overall survival; PFS, progression-free survival, SOC, standard of care.

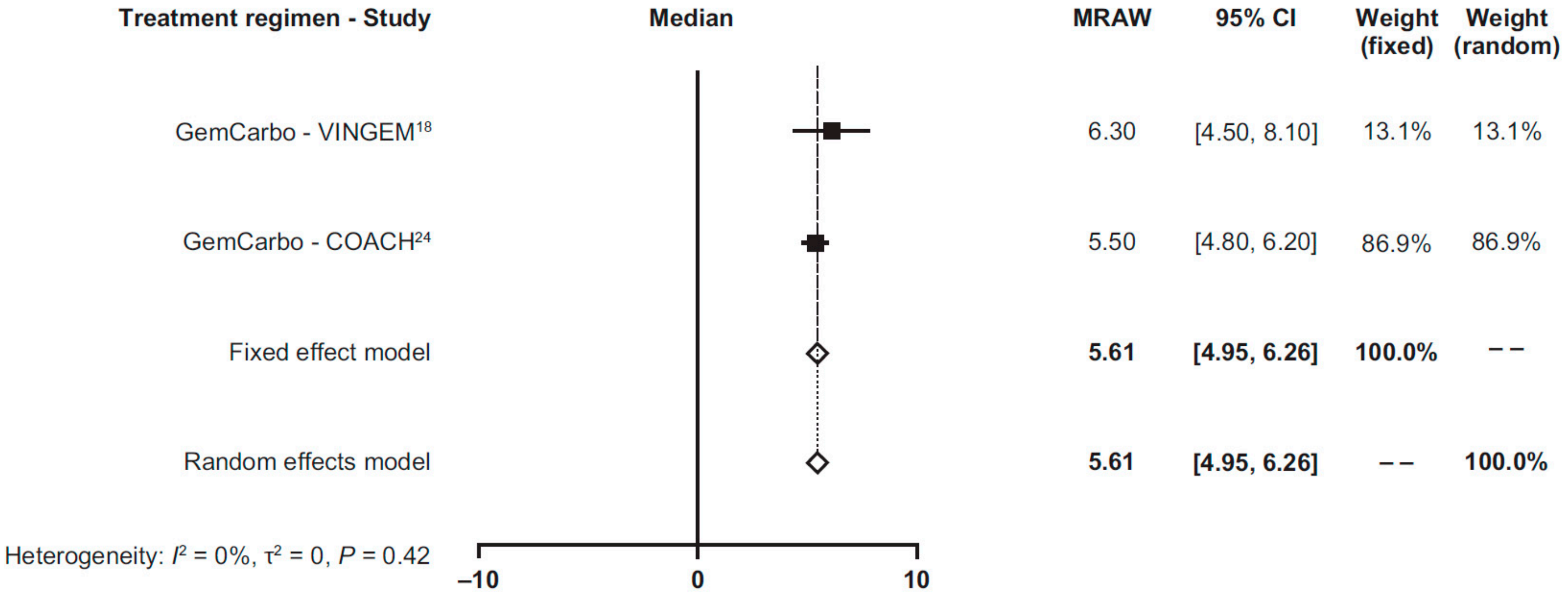

Supplementary Figure S7. Median PFS for SOC (GemCarbo) in the overall cis-ineligible networks.

References = [18,24]

CI, confidence interval; Cis, cisplatin; GemCarbo, gemcitabine + carboplatin; MRAW, raw or untransformed mean; PFS, progression-free survival, SOC, standard of care.

Supplementary Table S1. Cochrane risk-of-bias tool for randomized trials (RoB2).

Clinical Trial (Author Year)	Domain 1: Risk of bias arising from randomization process	Domain 2: Risk of bias due to deviations from the intended interventions	Domain 3: Missing outcome data	Domain 4: Risk of bias in measurement of the outcome	Domain 5: Risk of bias in selection of the reported results	Overall Risk
COACH (Park 2020) [24]	Low	Low	Low	Low	Low	Low
DANUBE (Powles 2020) [10]	Low	Low	Low	Low	Low	Low
EORTC 30924 (Sternberg 2006) [28]	Low	Low	Low	Low	Low	Low
EORTC 30986 (De Santis 2012) [29]	Low	Low	Low	Low	Some	Some
EORTC 30987 (Bellmunt 2012) [37]	High	Low	Low	Low	Low	High
GETUG V01 (Culine 2011) [30]	Low	Low	Low	Low	Low	Low
HE 16/03 (Bamias 2013) [25]	Some	High	Low	Low	Some	High
IMvigor130 (Galsky 2020) [9]	Low	Some	Low	Low	Low	Some
JASINT1 (De Santis 2016) [31]	High	Low	Low	Low	Some	High
KEYNOTE-361 (Powles 2021) [12]	Low	Low	Low	Low	Low	Low
Bamias 2004 [32]	Some	Low	Low	Low	Some	Some
Dogliotti 2007 [33]	High	Low	Low	Low	Low	High
Dreicer 2004 [34]	Some	Low	Low	Low	Low	Some
Lorusso 2005 [35]	High	Low	Low	Low	Low	High
Siefker-Radke 2002 [36]	High	Low	Low	Low	Low	High
von der Maase 2005 [16]	High	Low	Low	Low	Low	High
VINGEM (Holmsten 2020) [18]	Some	Low	Low	Low	Low	Some

Supplementary Table S2. Overview of studies in each network.

Study Name (Citation)	Treatment regimen	N	ORR, %	mPFS, months (95% CI)	mOS. months (95% CI)
Cisplatin-Eligible/Mixed					
KEYNOTE-361 (Alva 2020) [8]	Pembro + GemPlat	351	55	8.3 (7.5, 8.5)	17.0 (14.5, 19.5)
	Pembro	307	30	-	15.6 (12.1, 17.9)
	GemPlat	352	45	7.1 (6.4, 7.9)	14.3 (12.3, 16.7)
IMvigor130 (Galsky 2020; Davis 2021) [9, 13]	Atezo + GemPlat	451	48	8.2 (6.5, 8.3)	16.1 (14.2, 18.8)
	Atezo	362	23	-	15.2 (13.1, 17.7)
	GemPlat	400	45	6.3 (6.2, 7.0)	13.4 (11.9, 15.2)
DANUBE (Powles 2020) [10]	DUR	346	26	2.3 (1.9, 3.5)	13.2 (10.3, 15.0)
	DUR + TREME	342	36	3.7 (3.4, 3.8)	15.1 (13.1, 18.0)
	GemPlat	344	49	6.7 (5.7, 7.3)	12.1 (10.9, 14.0)
HE 16/03 (Bamias 2013) [25]	ddMVAC	63	60	-	-
	ddGemCis	63	65	-	-
EORTC 30987 (Bellmunt 2012) [37]	GemCis + PAX	312	55	-	15.8 (13.6, 17.5)
	GemCis	314	44	-	12.7 (11.0, 14.4)
EORTC 30924 (Sternberg 2006) [28]	ddMVAC	134	72	-	-
	MVAC	129	58	-	-
Dreicer 2004 [34]	MVAC	44	28	-	-
	Carbo + PAX	41	28	-	-
Bamias 2004 [32]	MVAC	109	54	9.4 (7.4, 11.3)	14.2 (12.5, 15.9)
	Cis + DXT	111	37	6.1 (5.6, 6.6)	9.3 (12.5, 15.9)
Lorusso 2005 [35]	GemCis + PAX	42	43	-	-
	GemCis	43	44	-	-
von der Maase 2005 [16]	MVAC	202	46	8.3 (7.3, 9.7)	14.1 (12.3, 15.5)
	GemCis	203	49	7.7 (6.8, 8.8)	15.2 (13.2, 17.3)
Dogliotti 2007 [33]	GemCis	55	66	-	-
	GemCarbo	55	56	-	-
Cisplatin-Ineligible (Wide)					
KEYNOTE-361 (Powles 2021) [39]	Pembro + GemCarbo	195	47	-	-
	Pembro	170	28	-	14.6 (10.2, 17.9)
	GemCarbo	196	42	-	12.3 (10, 15.5)
IMvigor130 (Galsky 2020) [9]	Atezo + GemCarbo	311	48	-	14.3 (12.0, 16.5)
	Atezo	190	-	-	-
	GemCarbo	183	42	-	13.0 (10.6, 15.2)

Study Name (Citation)	Treatment regimen	N	ORR, %	mPFS, months (95% CI)	mOS. months (95% CI)
DANUBE (Powles 2020) [10]	DUR + TREME	158	36	-	-
	DUR	149	24	-	-
	GemPlat	151	46	-	-
VINGEM (Holmsten 2020) [18]	VFL + Gem	32	63	6.2 (4.4, 8.3)	12.5 (8.4. 15.8)
	GemCarbo	30	40	6.3 (4.2, 7.8)	10.7 (7.4, 17.0)
JASINT1 (De Santis 2016) [31]	VFL + Carbo	35	44	5.9 (4.2, 9.4)	14.0 (8.3, 20.1)
	VFL + Gem	34	29	6.1 (4.6, 10.4)	12.8 (9.5, 17.7)
EORTC 30986 (De Santis 2012) [29]	M-CAVI	119	30	-	-
	GemCarbo	119	42	-	-
GETUG V01 (Culine 2011) [30]	Gem + OXP	22	27	-	8.1 (3.7, 10.7)
	Gem	22	43	-	5.4 (3.3, 13.4)
COACH (Park 2020) [24]	Gem + OXP	40	55	4.4 (2.7, 6.1)	11.0 (0.9, 15.0)
	Gem + Carbo	39	49	5.5 (4.8, 6.2)	9.1 (5.2, 13.0)
Cisplatin-Ineligible (Strict)					
DANUBE (Powles 2020) [10]	DUR + TREME	158	36	-	-
	DUR	149	24	-	-
	GemPlat	151	46	-	-
VINGEM (Holmsten 2020) [18]	VFL + Gem	32	63	6.2 (4.4, 8.3)	12.5 (8.4. 15.8)
	GemCarbo	30	40	6.3 (4.2, 7.8)	10.7 (7.4, 17.0)
JASINT1 (De Santis 2016) [31]	VFL + Carbo	35	44	5.9 (4.2, 9.4)	14.0 (8.3, 20.1)
	VFL + Gem	34	29	6.1 (4.6, 10.4)	12.8 (9.5, 17.7)
EORTC 30986 (De Santis 2012) [29]	M-CAVI	119	30	-	-
	GemCarbo	119	42	-	-
GETUG V01 (Culine 2011) [30]	Gem + OXP	22	27	-	8.1 (3.7, 10.7)
	Gem	22	43	-	5.4 (3.3, 13.4)
COACH (Park 2020) [24]	Gem + OXP	40	55	4.4 (2.7, 6.1)	11.0 (0.9, 15.0)
	GemCarbo	39	49	5.5 (4.8, 6.2)	9.1 (5.2, 13.0)

Atezo, atezolizumab; Carbo, carboplatin; Cis, cisplatin; CI, confidence interval; ddGemCis, dose-dense gemcitabine + cisplatin; ddMVAC, dose-dense methotrexate + vinblastine + doxorubicin + cisplatin; DUR, durvalumab; DXT, docetaxel; Gem, gemcitabine; GemCarbo, gemcitabine + carboplatin; GemCis, gemcitabine + cisplatin; GemPlat, gemcitabine + platinum (cisplatin or carboplatin); M-CAVI. methotrexate + carboplatin + vinblastine; mOS, median overall survival; mPFS, median progression-free survival; MVAC, methotrexate + vinblastine + doxorubicin + cisplatin; ORR, overall response rate; OXP, oxaliplatin; PAX, paclitaxel; Pembro, pembrolizumab; TREME, tremelimumab; VFL, vinflunine.

Supplementary Table S3. League table of relative treatment effects on OS (HR, Crl). Cis-eligible/mixed network.

	ddMVAC	ddGemCis	Atezo + GemPlat	DUR + TREME	PAX + GemPlat	Pembro + GemPlat	MVAC	Pembro	Atezo	DUR	GemPlat	DXT + Cis
ddMVAC		0.94 (0.61, 1.43)	1.20 (0.82, 1.75)	1.22 (0.83, 1.77)	1.22 (0.83, 1.79)	1.23 (0.84, 1.80)	**1.31** (1.01, 1.72)	1.32 (0.90, 1.93)	1.42 (0.96, 2.07)	1.42 (0.97, 2.07)	**1.43** (1.02, 2.01)	**2.00** (1.33, 3.03)
ddGemCis	1.06 (0.70, 1.63)		1.28 (0.73, 2.26)	1.29 (0.73, 2.29)	1.29 (0.73, 2.29)	1.31 (0.74, 2.31)	1.40 (0.84, 2.32)	1.40 (0.79, 2.47)	1.50 (0.85, 2.66)	1.50 (0.86, 2.66)	1.52 (0.89, 2.63)	**2.11** (1.17, 3.86)
Atezo + GemPlat	0.83 (0.57, 1.21)	0.78 (0.44, 1.37)		1.01 (0.79, 1.29)	1.01 (0.79, 1.29)	1.02 (0.80, 1.30)	1.09 (0.84, 1.43)	1.09 (0.85, 1.41)	1.18 (0.99, 1.40)	1.18 (0.92, 1.50)	**1.19** (1.00, 1.41)	**1.66** (1.10, 2.52)
DUR + TREME	0.82 (0.56, 1.20)	0.77 (0.44, 1.37)	0.99 (0.77, 1.26)		1.00 (0.78, 1.28)	1.01 (0.79, 1.29)	1.08 (0.82, 1.41)	1.08 (0.84, 1.39)	1.16 (0.91, 1.50)	1.16 (0.98, 1.39)	1.18 (0.99, 1.40)	**1.64** (1.08, 2.48)
PAX + GemPlat	0.82 (0.56, 1.20)	0.77 (0.44, 1.36)	0.99 (0.77, 1.26)	1.00 (0.78, 1.28)		1.01 (0.79, 1.30)	1.08 (0.82, 1.42)	1.08 (0.84, 1.39)	1.16 (0.91, 1.50)	1.16 (0.91, 1.49)	1.18 (0.99, 1.40)	**1.64** (1.09, 2.49)
Pembro + GemPlat	0.81 (0.56, 1.19)	0.76 (0.43, 1.34)	0.98 (0.77, 1.25)	0.99 (0.77, 1.26)	0.99 (0.77, 1.26)		1.07 (0.81, 1.40)	1.07 (0.89, 1.28)	1.15 (0.90, 1.48)	1.15 (0.90, 1.47)	1.16 (0.98, 1.38)	**1.62** (1.07, 2.45)
MVAC	**0.76** (0.58, 0.99)	0.72 (0.43, 1.18)	0.92 (0.70, 1.19)	0.93 (0.71, 1.22)	0.93 (0.70, 1.22)	0.94 (0.72, 1.23)		1.00 (0.76, 1.32)	1.08 (0.82, 1.42)	1.08 (0.83, 1.42)	1.09 (0.89, 1.34)	**1.52** (1.11, 2.08)
Pembro	0.76 (0.52, 1.12)	0.72 (0.40, 1.26)	0.91 (0.71, 1.17)	0.92 (0.72, 1.19)	0.93 (0.72, 1.19)	0.94 (0.78, 1.12)	1.00 (0.76, 1.31)		1.08 (0.83, 1.39)	1.08 (0.84, 1.38)	1.09 (0.91, 1.30)	**1.52** (1.00, 2.30)
Atezo	0.71 (0.48, 1.04)	0.67 (0.38, 1.18)	0.85 (0.71, 1.01)	0.86 (0.67, 1.10)	0.86 (0.67, 1.10)	0.87 (0.68, 1.12)	0.93 (0.70, 1.23)	0.93 (0.72, 1.20)		1.00 (0.78, 1.29)	1.01 (0.84, 1.21)	1.41 (0.93, 2.15)
DUR	0.71 (0.48, 1.03)	0.67 (0.38, 1.17)	0.85 (0.67, 1.08)	0.86 (0.72, 1.02)	0.86 (0.67, 1.10)	0.87 (0.68, 1.11)	0.93 (0.71, 1.21)	0.93 (0.72, 1.19)	1.00 (0.78, 1.28)		1.01 (0.85, 1.20)	1.41 (0.93, 2.13)
GemPlat	**0.70** (0.50, 0.98)	0.66 (0.38, 1.12)	**0.84** (0.71, 1.00)	0.85 (0.71, 1.01)	0.85 (0.71, 1.01)	0.86 (0.72, 1.02)	0.92 (0.75, 1.13)	0.92 (0.77, 1.10)	0.99 (0.83, 1.18)	0.99 (0.84, 1.17)		1.39 (0.96, 2.03)
DXT + Cis	**0.50** (0.33, 0.75)	**0.47** (0.26, 0.85)	**0.60** (0.40, 0.91)	**0.61** (0.40, 0.93)	**0.61** (0.40, 0.92)	**0.62** (0.41, 0.93)	**0.66** (0.48, 0.90)	**0.66** (0.44, 1.00)	0.71 (0.47, 1.07)	0.71 (0.47, 1.08)	0.72 (0.49, 1.04)	

**indicates statistical significance.

A fixed effect model was used in this analysis as heterogeneity between studies was acceptable according to Cochrane's Q and Higgin's I2 criteria. [11]

Atezo, atezolizumab; Cis, cisplatin; CrI, credible interval; ddGemCis, dose-dense gemcitabine + cisplatin; ddMVAC, dose-dense methotrexate + vinblastine + doxorubicin + cisplatin; DUR, durvalumab; DXT, docetaxel; GemPlat, gemcitabine + platinum (cisplatin or carboplatin); HR, hazard ratio; MVAC, methotrexate + vinblastine + doxorubicin + cisplatin; OS, overall survival; PAX, paclitaxel; Pembro, pembrolizumab; TREME, tremelimumab.

Supplementary Table S4. League table of relative treatment effects on OS (HR, Crl). Cis-ineligible (wide) network.

	Pembro	Pembro + GemCis	Atezo + GemCis	Atezo	VFL + Gem	GemCis	OXP + Gem
Pembro		1.01 (0.79)	1.10 (0.80, 1.50)	1.17 (0.83, 1.64)	1.30 (0.69, 2.46)	1.21 (0.94, 1.54)	1.68 (0.97, 2.89)
Pembro + GemCis	0.99 (0.78, 1.26)		1.08 (0.80, 1.47)	1.15 (0.83, 1.61)	1.28 (0.69, 2.41)	1.19 (0.94, 1.50)	1.66 (0.95, 2.83)
Atezo + GemCis	0.91 (0.67, 1.25)	0.92 (0.68, 1.25)		1.07 (0.85, 1.34)	1.18 (0.64, 2.20)	1.10 (0.90, 1.34)	1.53 (0.90, 2.58)
Atezo	0.85 (0.61, 1.20)	0.87 (0.62, 1.21)	0.94 (0.75, 1.18)		1.11 (0.59, 2.09)	1.03 (0.81, 1.31)	1.43 (0.83, 2.47)
VFL + Gem	0.77 (0.41, 1.45)	0.78 (0.41, 1.46)	0.85 (0.45, 1.55)	0.90 (0.48, 1.68)		0.93 (0.51, 1.65)	1.29 (0.60, 2.75)
GemCis	0.83 (0.65, 1.06)	0.84 (0.67, 1.06)	0.91 (0.74, 1.11)	0.97 (0.76, 1.23)	1.07 (0.61, 1.94)		1.39 (0.85, 2.26)
OXP + Gem	0.60 (0.35, 1.04)	0.60 (0.35, 1.05)	0.65 (0.39, 1.11)	0.70 (0.41, 1.21)	0.77 (0.36, 1.68)	0.72 (0.44, 1.18)	

A fixed effect model was used in this analysis as heterogeneity between studies was acceptable according to Cochrane's Q and Higgin's I2 criteria. [11]

Atezo, atezolizumab; Cis, cisplatin; CrI, credible interval; Gem, gemcitabine; GemCis, gemcitabine + cisplatin; HR, hazard ratio; MVAC, methotrexate + vinblastine + doxorubicin + cisplatin; OS, overall survival; OXP, oxaliplatin; Pembro, pembrolizumab; VFL, vinflunine.

Supplementary Table S5. League table of relative treatment effects on OS (HR, Crl). Cis-ineligible (strict) network.

	DUR + TREME	DUR	GemCis	VFL + Gem	OXP + Gem
DUR + TREM		1.08 (0.84, 1.39)	1.16 (0.90, 1.49)	1.26 (0.66, 2.35)	1.61 (0.93, 2.79)
DUR	0.92 (0.72, 1.19)		1.07 (0.83, 1.38)	1.16 (0.61, 2.18)	1.49 (0.85, 2.57)
GemCis	0.86 (0.67, 1.11)	0.93 (0.72, 1.20)		1.08 (0.60, 1.92)	1.39 (0.85, 2.26)
VFL + Gem	0.80 (0.43, 1.51)	0.86 (0.46, 1.63)	0.93 (0.52, 1.66)		1.29 (0.61, 2.75)
OXP + Gem	0.62 (0.36, 1.08)	0.67 (0.39, 1.17)	0.72 (0.44, 1.18)	0.78 (0.36, 1.64)	

A fixed effect model was used in this analysis as heterogeneity between studies was acceptable according to Cochrane's Q and Higgin's I2 criteria. [11]
Cis, cisplatin; CrI, credible interval; DUR, durvalumab; Gem, gemcitabine; GemCis, gemcitabine + cisplatin; HR, hazard ratio; OS, overall survival; OXP, oxaliplatin; TREME, tremelimumab; VFL, vinflunine.

Supplementary Table S6. League table of relative treatment effects on PFS (HR, Crl), Cis-eligible/mixed.

	ddGemCis	ddMVAC	Pembro + GemPlat	Atezo + GemPlat	DUR + TREME	PAX + GemPlat	MVAC	GemPlat	Pembro	DXT + Cis
ddGemCis		1.27 (0.82, 1.96)	1.47 (0.82, 2.59)	1.55 (0.87, 2.71)	1.62 (0.91, 2.84)	1.64 (0.92, 2.88)	**1.73** (1.04, 2.87)	**1.88** (1.09, 3.22)	**2.48** (1.39, 4.41)	**2.99** (1.61, 5.46)
ddMVAC	0.79 (0.51, 1.22)		1.16 (0.79, 1.69)	1.22 (0.84, 1.76)	1.28 (0.88, 1.86)	1.29 (0.89, 1.87)	**1.37** (1.05, 1.78)	**1.49** (1.07, 2.08)	**1.96** (1.34, 2.87)	**2.37** (1.54, 3.60)
Pembro + GemPlat	0.68 (0.39, 1.21)	0.86 (0.59, 1.26)		1.05 (0.83, 1.33)	1.10 (0.87, 1.40)	1.12 (0.87, 1.43)	1.18 (0.90, 1.54)	**1.28** (1.07, 1.53)	**1.69** (1.41, 2.03)	**2.04** (1.33, 3.14)
Atezo + GemPlat	0.65 (0.37, 1.15)	0.82 (0.57, 1.19)	0.95 (0.75, 1.21)		1.05 (0.84, 1.31)	1.06 (0.85, 1.33)	1.12 (0.86, 1.45)	**1.22** (1.04, 1.43)	**1.60** (1.26, 2.05)	**1.94** (1.26, 2.95)
DUR + TREME	0.62 (0.35, 1.09)	0.78 (0.54, 1.13)	0.91 (0.71, 1.16)	0.95 (0.76, 1.20)		1.01 (0.81, 1.27)	1.07 (0.82, 1.39)	1.16 (0.99, 1.37)	**1.53** (1.20, 1.96)	**1.85** (1.21, 2.81)
PAX + GemPlat	0.61 (0.35, 1.09)	0.77 (0.53, 1.12)	0.90 (0.70, 1.15)	0.94 (0.75, 1.18)	0.99 (0.79, 1.24)		1.06 (0.81, 1.37)	1.15 (0.98, 1.35)	**1.51** (1.19, 1.95)	**1.83** (1.19, 2.80)
MVAC	**0.58** (0.35, 0.97)	**0.73** (0.56, 0.96)	0.85 (0.65, 1.11)	0.89 (0.69, 1.16)	0.94 (0.72, 1.21)	0.95 (0.73, 1.23)		1.09 (0.89, 1.34)	**1.44** (1.09, 1.89)	**1.73** (1.23, 2.42)
GemPlat	**0.53** (0.31, 0.92)	**0.67** (0.48, 0.94)	**0.78** (0.65, 0.93)	**0.82** (0.70, 0.96)	0.86 (0.73, 1.01)	0.87 (0.74, 1.02)	0.92 (0.75, 1.13)		**1.32** (1.10, 1.59)	**1.59** (1.07, 2.35)
Pembro	**0.40** (0.23, 0.72)	**0.51** (0.35, 0.75)	**0.59** (0.49, 0.71)	**0.62** (0.49, 0.79)	**0.65** (0.51, 0.83)	**0.66** (0.51, 0.84)	**0.70** (0.53, 0.92)	**0.76** (0.63, 0.91)		1.20 (0.78, 1.86)
DXT + Cis	**0.33** (0.18, 0.62)	**0.42** (0.28, 0.65)	**0.49** (0.32, 0.75)	**0.52** (0.34, 0.79)	**0.54** (0.36, 0.83)	**0.55** (0.36, 0.84)	**0.58** (0.41, 0.81)	**0.63** (0.43, 0.94)	0.83 (0.54, 1.28)	

**indicates statistical significance.A fixed effect model was used in this analysis as heterogeneity between studies was acceptable according to Cochrane's Q and Higgin's I2 criteria. [11]

Atezo, atezolizumab; Cis, cisplatin;; ddGemCis, dose-dense gemcitabine + cisplatin; ddMVAC, dose-dense methotrexate + vinblastine + doxorubicin + cisplatin; DUR, durvalumab; DXT, docetaxel; GemPlat, gemcitabine + platinum (cisplatin or carboplatin); HR, hazard ratio; PAX, paclitaxel; Pembro, pembrolizumab; PFS, progression-free survival; TREME, tremelimumab.

Supplementary Table S7. League table of relative treatment effects on PFS (HR, CrI). Cis-ineligible (wide) network.

	VFL + Gem	Atezo + GemCis	Pembro + GemCis	M-CAVI	GemCis	OXP + Gem
VFL + Gem		1.11 (0.64, 1.95)	1.14 (0.64, 2.03)	1.28 (0.71, 2.29)	1.33 (0.78, 2.26)	1.44 (0.70, 2.96)
Atezo + GemCis	0.90 (0.51, 1.57)		1.02 (0.76, 1.39)	1.14 (0.83, 1.57)	1.19 (0.99, 1.44)	1.30 (0.77, 2.17)
Pembro + GemCis	0.88 (0.49, 1.57)	0.98 (0.72, 1.32)		1.12 (0.79, 1.59)	1.17 (0.92, 1.48)	1.27 (0.74, 2.16)
M-CAVI	0.78 (0.44, 1.41)	0.87 (0.64, 1.20)	0.89 (0.63, 1.27)		1.04 (0.80, 1.35)	1.13 (0.66, 1.94)
GemCis	0.75 (0.44, 1.28)	0.84 (0.70, 1.01)	0.86 (0.68, 1.09)	0.96 (0.74, 1.25)		1.09 (0.67, 1.76)
OXP + Gem	0.69 (0.34, 1.42)	0.77 (0.46, 1.30)	0.79 (0.46, 1.35)	0.88 (0.51, 1.53)	0.92 (0.57, 1.49)	

A fixed effect model was used in this analysis as heterogeneity between studies was acceptable according to Cochrane's Q and Higgin's I2 criteria. [11]
Atezo, atezolizumab; Cis, cisplatin; Gem, gemcitabine; GemCis, gemcitabine + cisplatin; HR, hazard ratio; M-CAVI, methotrexate + carboplatin + vinblastine; OXP, oxaliplatin; Pembro, pembrolizumab; PFS, progression-free survival; OXP, oxaliplatin; TREME. tremelimumab; VFL, vinflunine.

Supplementary Table S8. League table of relative treatment effects on PFS (HR, CrI). Cis-ineligible (strict) network.

	VFL + Gem	M-CAVI	GemCis	OXP + Gem
VFL + Gem		1.28 (0.70, 2.33)	1.33 (0.78, 2.27)	1.45 (0.70, 2.95)
M-CAVI	0.78 (0.43, 1.42)		1.04 (0.80, 1.36)	1.13 (0.66, 1.96)
GemCis	0.75 (0.44, 1.28)	0.96 (0.74, 1.25)		1.09 (0.67, 1.76)
OXP + Gem	0.69 (0.34, 1.42)	0.88 (0.51, 1.52)	0.92 (0.57, 1.48)	

A fixed effect model was used in this analysis as heterogeneity between studies was acceptable according to Cochrane's Q and Higgin's I2 criteria. [11]
Cis, cisplatin; GemCis, gemcitabine + cisplatin; Gem, gemcitabine; HR, hazard ratio; M-CAVI, methotrexate + carboplatin + vinblastine; OXP, oxaliplatin; PFS, progression-free survival; VFL, vinflunine.
